# Evaluation of True Bonding Strength for Adhesive Bonded Carbon Fiber-Reinforced Plastics

**DOI:** 10.3390/ma17020394

**Published:** 2024-01-12

**Authors:** Maruri Takamura, Minori Isozaki, Shinichi Takeda, Yutaka Oya, Jun Koyanagi

**Affiliations:** 1 Department of Materials Science and Technology, Tokyo University of Science, Tokyo 125-8585, Japan; 8217056@alumni.tus.ac.jp (M.T.); 8219012@alumni.tus.ac.jp (M.I.); oya@rs.tus.ac.jp (Y.O.); 2 Japan Aerospace Exploration Agency, Tokyo 181-0015, Japan; takeda.shinichi@jaxa.jp

**Keywords:** numerical simulation, bond strength test, interfacial strength, CFRP ultrasonic welding

## Abstract

Carbon fiber-reinforced thermoplastics (CFRTPs) have attracted attention in aerospace because of their superior specific strength and stiffness. It can be assembled by adhesive bonding; however, the existing evaluation of bonding strength is inadequate. For example, in a single-lap shear test, the weld zone fails in a combined stress state because of the bending moment. Therefore, the strength obtained experimentally is only the apparent strength. The true bonding strength was obtained via numerical analysis by outputting the local stress state at the initiation point of failure. In this study, the apparent and true bonding strengths were compared with respect to three types of strength evaluation tests to comprehensively evaluate bonding strength. Consequently, the single-lap shear test underestimates the apparent bonding strength by less than 14% of the true bonding strength. This indicates that care should be taken when determining the adhesion properties for use in numerical analyses based on experimental results. We also discussed the thickness dependence of the adhesive on the stress state and found that the developed shear test by compression reduced the discrepancy between apparent and true strength compared with the single-lap shear test and reduced the thickness dependence compared with the flatwise tensile test.

## 1. Introduction

Carbon fiber-reinforced plastics (CFRPs) have attracted attention in the automotive and aerospace industries because of their superior specific strength and stiffness [1,2,3]. For example, its light weight and high strength improve the fuel efficiency of automobiles and aircraft, thereby reducing their environmental impact. Among CFRPs, those whose resin portion is made of thermoplastic resin are called carbon fiber-reinforced thermoplastics (CFRTPs). CFRTP has advantages over CFRP, which is made of thermoplastic resin, including easier molding and recycling. In addition, CFRTPs can be assembled by welded joints because they melt when heated. In contrast to fastening with bolts, welding eliminates the need for bolt drilling, resulting in weight reduction and improving mechanical properties by reducing stress concentrations [4,5,6].

Once the weld joining of CFRTPs is completed, the strength of these joints needs to be verified to evaluate the strength of the joints and design the structure using numerical simulations. For example, the single-lap shear (SLS) and flatwise tensile (FW) tests are known strength evaluation methods for bonded materials [7,8]. SLS evaluates the strength of a joint by applying shear stress to bonded surfaces, whereas FW evaluates the strength of a joint by applying tensile stress to bonded surfaces. Both are often used in bond strength tests. However, they have the following problems. The stress state at the bonded interface depends on the specimen size [9,10,11,12,13,14]. The bond fails under the combined stress state [15,16,17,18,19]. The fracture stress obtained by dividing the experimental reaction force at failure by the bonded area is the average apparent strength, and if this apparent strength is used as the strength of the interface in the numerical analysis as it is, the experiment cannot be reproduced correctly [20,21]. The size of the specimens must be large to comply with test standards such as ISO and ASTM, and the joints are prone to combined stresses and expensive to test.

To address these problems, we must use numerical analysis to investigate the stress state at the initiation point of failure in strength evaluation tests and perform a comprehensive evaluation to identify the apparent strength obtained experimentally and to what extent it reproduces the true strength. The stresses applied to the interface during the strength evaluation testing are distributed isotropically, and it is difficult to apply pure shear or normal stress to bonded CFRTP [22,23,24,25,26,27,28,29]. However, the nominal stress, which is obtained by dividing the breaking load by the bonded area, assumes that the stress distribution is uniform. Therefore, the obtained fracture stress is a weaker estimate than the strength of the interface for pure shear or normal stress. The apparent strength is referred to as the stress at failure, which is the experimental reaction force at failure divided by the weld area, and the localized failure stress at the initiation point of failure is referred to as the true strength. In a previous study, DeVries et al. discussed the stress state in SLS from a material-mechanics perspective [30]. Villegas et al. demonstrated that in SLS, the bond strength at the edge of the bonded surface directly affects the strength of the entire interface [31]. Redmann et al. proposed a block shear test method to reduce these effects [32].

The following is the background of our research: We have been conducting a combined experimental and analytical study on the ultrasonic welding of CFRTP and investigated the effects of temperature increase and adhesive shape during welding [33]. Ultrasonic welding is a method of welding materials by bringing a metal transducer into contact with overlapping adherends and propagating ultrasonic vibrations of around 20 kHz while applying pressure. The ultrasonic vibration is a longitudinal vibration perpendicular to the welding surface, and the frictional heat generated by the vibration between materials and molecules raises the temperature and melts the thermoplastic resin. Welding can be performed only by applying ultrasonic vibration for a short period of time of 5 s or less under ambient temperature and pressure, making it a highly efficient and low-energy joining method. Generally, when ultrasonic welding is used, a thermoplastic resin called an energy director is placed between the adherends as an adhesive to serve as the starting point for melting, thereby enabling stable welding. In ultrasonic welding, rapid temperature changes occur in a short period of time, so various parameters, such as ultrasonic frequency, welding time, and welding pressure, are complicated and affect the welding conditions [34,35,36,37,38,39]. Therefore, it is important to select these parameters appropriately. Hence, specimens after ultrasonic welding need to be carefully evaluated. In addressing this issue, we noticed that the existing strength evaluation test methods did not adequately evaluate the adhesive strength. Thus, we embarked on a comprehensive evaluation of bond strength using numerical simulation aiming to predict the local true joint strength from the experimentally obtained apparent strength of ultrasonically welded CFRTP.

In this study, by comparing the true joint interface strength with the apparent strength, we suggest the dangers of existing strength evaluation tests and provide guidelines for a comprehensive interface strength evaluation method. We studied the relationship between the apparent strength in bond strength tests of welded specimens that failed at the adhesive–adherend bonding interface, and the true strength at the fracture initiation point was investigated using a numerical simulation of the stress state at the fracture initiation point. Based on these results, a fracture envelope was drawn to comprehensively evaluate the bond strength. In addition, we propose a novel shear test by compression that reduces the discrepancy between the apparent strength and true strength and is less dependent on the thickness of the adhesive. Through comparison of the apparent strength in this shear test by compression with that in the SLS and FW tests, respectively, the percentage of underestimation of apparent strength, the stress state at the actual fracture initiation point, and the effect of adhesive thickness on apparent strength were discussed.

## 2. Methods

### 2.1. Determination of Interfacial Strength between Adhesive and Adherend

In order to investigate the stress state at the bond interface at the onset of failure in various bond strength tests by numerical simulation, the value of the interfacial strength between the adhesive and the adherend was first determined. This interfacial strength was determined on an experimental basis, as reasonable values were used.

#### 2.1.1. Specimen Welding and Shear Test by Compression

**Figure 1:**
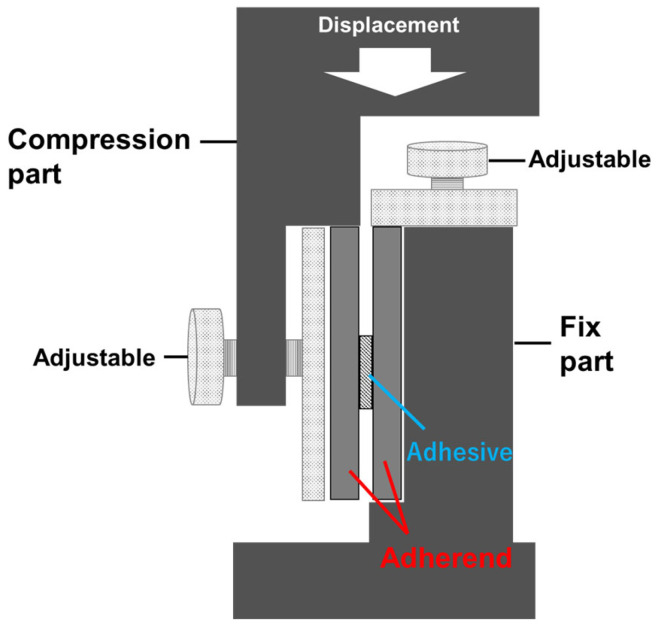
Illustration of the fixture developed for the shear test by compression. One side of the specimen is fixed with a fixed part, and the other side is compressed from the top of the specimen with a compression part to shear failure at the adhesive interface.

We developed a shear test using compression (SC) to conduct bond strength tests using small specimens. SC is a test method in which a bonded specimen is fractured by applying displacement in the shear direction to the bonded surface. This is similar to SLS; however, because SC applies a compressive load, whereas SLS applies a tensile load, the adherend does not require a certain length to hold the tensile jig in place. The use of smaller specimens reduces the bending moments and lowers the cost of the experiment. A schematic of the jig used for the SC, as shown in Figure 1, fixes one side of the adherend and compresses the other test piece from above.

In the welding experiment, quasi-isotropic polyether ether ketone reinforced with carbon fiber (CF/PEEK) (IMS/PEEK, Teijin, Osaka, Japan) with a thickness of 2 mm and a length of 30 × 30 cm per side was used as the adherend, and three layers of PEEK mesh (#25, Clever Co., Ltd., Toyohashi, Japan) were sandwiched between the adherends as an adhesive. The specimens were heated by applying 19.5 kHz ultrasonic waves for 3 s under a pressure of 0.1 MPa using an ultrasonic welding machine (JP80s, SEIDENSHA ELECTRONICS Co., LTD, Tokyo, Japan). A circular horn with a diameter of 10 mm was used as a transducer. After the ultrasonic vibration was unloaded, adhesion was performed by maintaining a holding load of 0.1 MPa for 5 s. Then, to confirm that the welding was completed, the condition of the adhesive in the welded area was observed by obtaining CT scan imaging of the welded specimen using a nondestructive inspection device (inspeXio MX-225CTS, SHIMADZU, Kyoto, Japan). A jig for SC was set up on a universal testing machine (Model 8802, INSTRON, Kawasaki, Japan) to evaluate the bond strengths of the welded specimens. SC was performed to obtain the reaction forces at failure, and the fracture surfaces of the specimens were observed using a Digital Microscope (VHX-6000, KEYENCE, Osaka, Japan).

#### 2.1.2. Numerical Analysis to Fit Cohesive Properties to Experimental Results

In numerical simulations, a parabolic criterion can be used as the failure criterion for the interface, as expressed in Equation (1) proposed by Ogihara et al. [40], where $Y_{n}$ is the pure normal strength of the interface, $Y_{s}$ is the pure shear strength of the interface, and $t_{n}$ and $t_{s}$ are the normal and shear stress at the interface, respectively. Here, normal stress indicates the vertical stress to the interface. When the value on the left-hand side reached one, the interface began to fail. Furthermore, Koyanagi et al. proved that the interfacial strength can be expressed by Equation (2) as the ratio of shear strength to normal strength [41].

(1) $$\frac{t_{n}}{Y_{n}}+{\left\{\frac{t_{s}}{Y_{s}}\right\}}^{2}=1$$

(2) $$\sqrt{2}Y_{n}=Y_{s}$$

Therefore, appropriate, cohesive properties can be obtained by numerically simulating the SC and fitting the interfacial strength at which the reaction force is equal to the experimental value. SC, like other bond strength tests, fails at the interface in a combined stress state, but by reproducing the experiment with a numerical model, the pure shear strength at the interface and normal strength can be predicted. The stress at debonding in the material properties of this cohesive element represents the true interfacial strength. We used Abaqus2020, a numerical analysis software, to simulate the model with the same dimensions as in the experiment, with cohesive elements introduced at the adhesive–adherend interface. Here the cohesive element is a sufficiently stiff offset element with a thickness of 0 mm. It represents the separation behavior of the interface depending on the stress state. The interface strength can be determined by specifying $Y_{n}$, the pure vertical strength, and $Y_{s}$, the pure shear strength. The properties of the materials are listed in Table 1. However, the cohesive property in this model is a fitting parameter. Here, for the material property anisotropy of CF/PEEK, E1 and E2 are assigned to the in-plane direction of the material and E3 to the material thickness direction. The area of the welded part was obtained through CT scanning of the welded specimen using a nondestructive inspection device (inspeXio SMX-225CTS, SHIMADZU, Kyoto, Japan) before the test. The cohesive properties were fitted such that the reaction force at the edge of the model at the onset of failure was equal to the experimental value according to the shear strength–normal strength ratio in Equation (2). In this analytical model, we focused only on the fracture initiation point and hypothesized that interface separation would be introduced, but no material damage would occur.

### 2.2. Numerical Analysis to Derive True and Apparent Bond Strength

Numerical simulations of CS, SLS, and FW were performed to investigate changes in the relationship between true interfacial strength and apparent strength across different test methods, with the adhesive thickness being consistent in each test. In addition, we investigated changes in the apparent strength in each test when the adhesive thickness varied.

#### 2.2.1. Comparison of Underestimation of Apparent Strength in Three Different Bond Strength Tests

**Figure 2:**
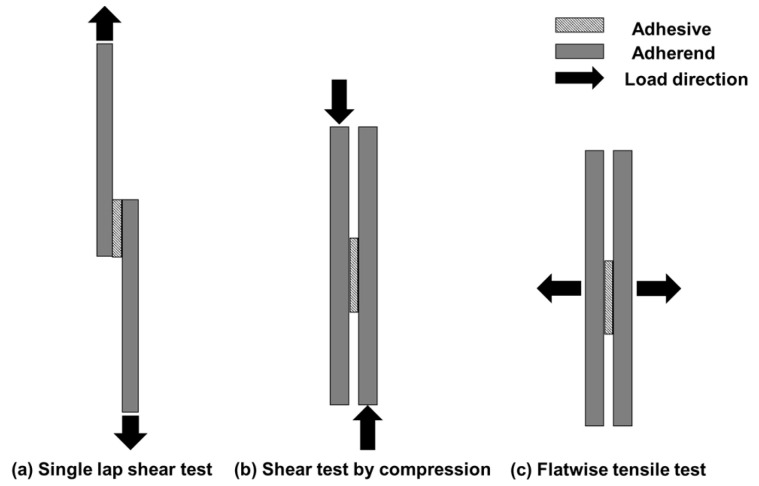
Schematic of the three types of bond strength tests discussed in this study. (**a**) Single-lap shear test and (**b**) shear test by compression apply load in shear direction to the interface, and (**c**) flatwise tensile test applies load vertically to the interface.

**Figure 3:**
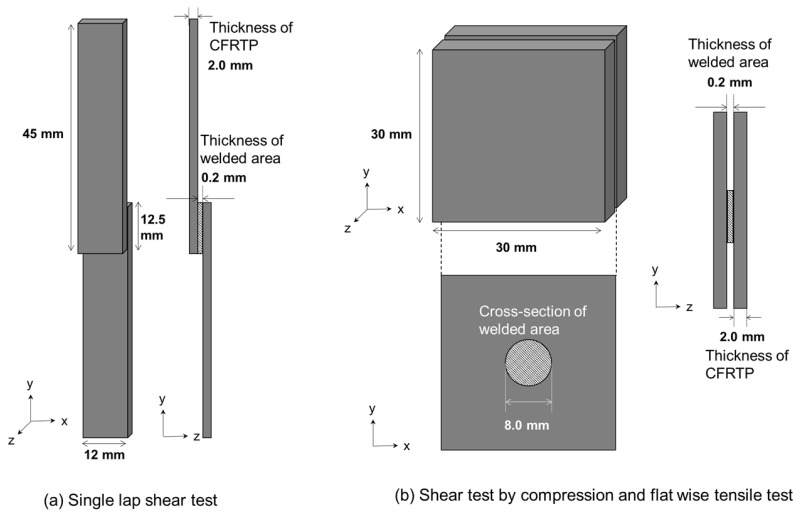
Dimensions and geometry of the simulation model. (**a**) Single-lap shear test was performed according to ISO standards (ISO 4587:2003). (**b**) Shear tests by compression and flatwise tensile tests gave different displacements for the same model.

Numerical simulations were performed for the three types of bond strength tests u sing the cohesive properties determined in the previous section, as shown in Figure 2. We modeled three types of tests: the ISO standard (ISO 4587:2003) [42] SLS, SC, and FW, which is a test with a different stress state at failure as a reference. The models were constructed using the cohesive elements introduced at the adhesive–adherend interface. As shown in Figure 3, the adhesive and adherend thicknesses were 0.2 mm and 2.0 mm, respectively, for all models. As for the mesh size dependence, the mesh-dependent effect due to stress concentration was reduced by filleting the edge of the welded area. Displacement in the shear direction to the adhesive surface was applied to the edge of the adherend in SLS and SC, and displacement in the normal direction in FW. Then, the stress state at the point of failure initiation and the apparent strength, obtained by dividing the reaction force at the start of fracture at the specimen end under displacement by the initial adhesive area, were calculated. A parabolic criterion was adopted as the destruction criterion. In these models, moreover, we focused only on the cases where the interface was the initiation point of fracture; thus, material fracture was not introduced. The properties of the materials are listed in Table 1.

#### 2.2.2. Effect of Adhesive Thickness on Stress State at Adhesive Interface in Three Different Bond Strength Tests

To discuss the dependence of the stress state in the bond strength test on the adhesive thickness, numerical simulations were used to compare thinner and thicker adhesive thicknesses, which were set at 0.2 mm in the last section. The apparent strengths of the three models (SLS, CS, and FW) were investigated when the adhesive thicknesses were changed to 0.1, 0.2, and 0.3 mm, respectively. Other dimensions were the same as the models in the previous chapter, as shown in Figure 3. The parabolic criterion adopted as the failure criterion was also the same as that adopted for the previous model, so the true interface strength is the same for all models. The properties of the materials are listed in Table 1.

## 3. Results and Discussion

### 3.1. Determination of Interfacial Strength between Adhesive and Adherend

**Figure 4:**
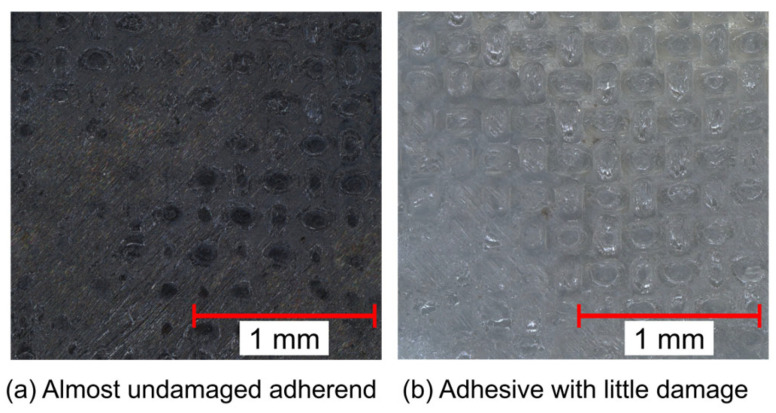
Digital microscope image of fracture surface after shear test using compression. There was little damage to (**a**) the adherend or (**b**) the adhesive, indicating that failure occurred at the interface between the adherend and the adhesive.

**Figure 5:**
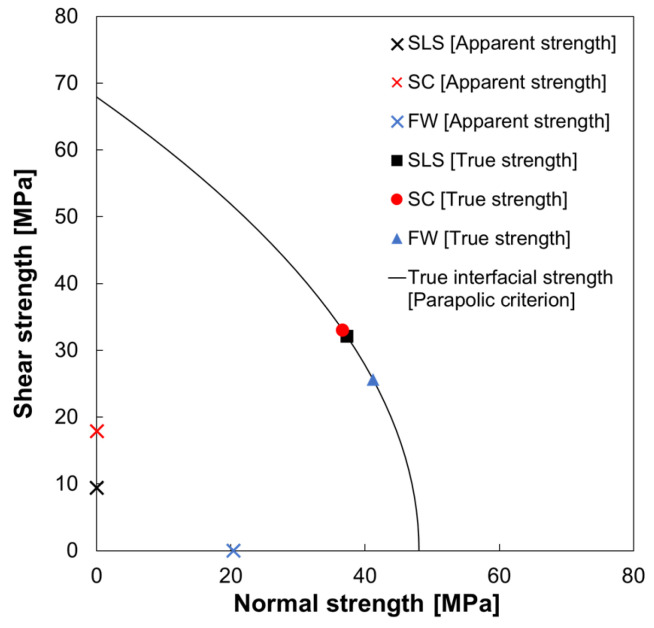
Local stress state at the fracture initiation point, which is the true interface strength. The average apparent strength is obtained through numerical simulations of SLS (single-lap shear test), SC (shear test using compression), and FW (flatwise tensile test), and the CFRP-polymer bonding interface strength (parabolic criterion) are plotted.

The CT scan of the welded specimen showed that the resin melted evenly in a circular pattern, with a welded area of approximately 50 mm^2^, and that the welding was complete. The reaction force at failure of the SC was obtained as 1.34 kN. Thus, the apparent strength was 26.8 MPa. The images of the fracture surface of the specimen taken with a digital microscope are shown in Figure 4, which shows that the adhesive and adherend showed little damage and that the specimen failed at the interface between the adhesive and the adherend. Therefore, it can be assumed that material failure of the adhesive does not occur when welded under the present welding conditions, and the fracture envelope can be drawn by focusing only on the interface between the adhesive and adherend. Based on the apparent strength obtained from the experimental results, the interface strength was fitted using a numerical simulation, and the cohesive properties listed in Table 1 were obtained. $Y_{n}$ and $Y_{s}$ were 48.0 and 67.9, respectively. The parabolic criterion was determined as the fracture envelope of Figure 5 according to this result and Equations (1) and (2).

### 3.2. Numerical Analysis to Derive True and Apparent Bond Strength

#### 3.2.1. Comparison of Underestimation of Apparent Strength in Three Different Bond Strength Tests

**Figure 6:**
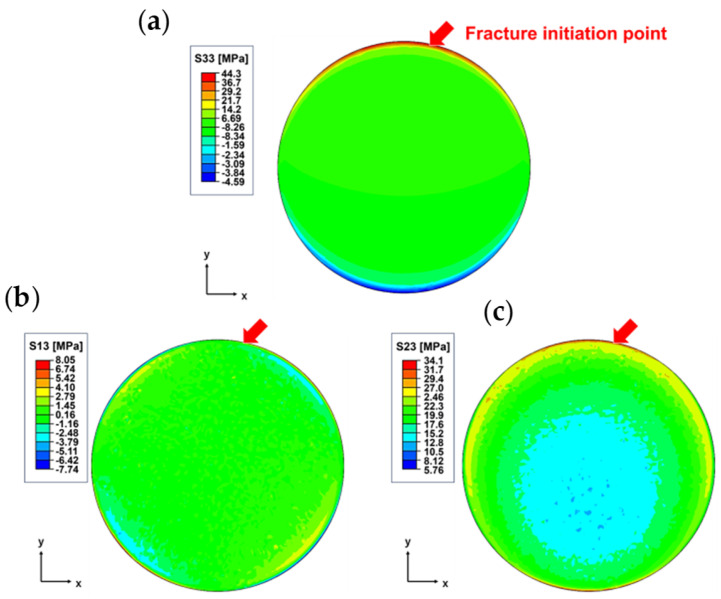
Numerical simulation results of cohesive elements in shear test by compression. (**a**) vertical stress S33, (**b**) transverse shear stress S13, and (**c**) longitudinal shear stress S23 are the output, and the break initiation point is indicated using arrows. There is a distribution of stress over the entire welded surface, indicating that tensile stress, in addition to shear stress, contributes to fracture.

The results of the numerical simulation outputting S33, S13, and S23 for the CS of the cohesive elements are shown in Figure 6. In this model, S33 represents the vertical stress against the adhesive–adherend interface, while S13 and S23 represent the shear stress. This indicates that not only shear stress but also tensile and compressive stresses are applied to the adhesive interface, indicating that tensile stresses contribute to the fracture at the fracture initiation point marked by the arrows in Figure 6. Numerical simulations of SLS, CS, and FW were performed, and the stress state at the failure initiation point is shown in Figure 5, with the shear stress on the vertical axis and the normal stress on the horizontal axis. The apparent strengths are also shown in Figure 5. The true interfacial strength was expressed using a parabolic criterion. Because the model is three-dimensional, only S33 is the normal stress for the cohesive elements, whereas transverse shear stress S13 and longitudinal shear stress S23 are the output of the shear stress. In this case, $\sqrt{S13^{2}+S23^{2}}$ was treated as the shear stress because the shear stress about the interface is expressed along one axis by coordinate transformation. The stress state at the fracture initiation point in the three tests lies in the line of the parabolic criterion used in this study, and it is clear from these plots in Figure 5 that the fracture is due to combined stresses. Focusing on the percentage of the combined stress state at the fracture initiation point for each bond strength test, we found that the $t_{n}/t_{s}$ percentage ratio for the SLS was approximately 54/46%. As SLS and CS are intended to fail only under shear stress, their respective apparent strengths $\left(Y_{n}^{App},\;Y_{s}^{App}\right)$ are plotted at (0, 9.39) and (0, 17.90) on the shear strength axis in Figure 5. A comparison of the true interface strength to the apparent strength shows that SLS underestimates by 13.8%, and SC underestimates by 26.4%. This underestimation occurs because the SLS and SC are subjected to a bending moment in addition to shear stress against the bonded surface, resulting in normal stress [43]. SC tends to have higher shear stresses at the interface than SLS because of the shorter longitudinal direction of the specimen in SLS, which suppresses rotation. The $t_{n}/t_{s}$ percentage ratio for FW was approximately 62/38%. As FW is intended for tensile failure, the apparent strength $\left(Y_{n}^{App},Y_{s}^{App}\right)$ was plotted at (20.4, 0) on the normal strength axis. The apparent strength underestimates the true interfacial strength by 42.4%, owing to the shear stress caused by the difference in Poisson’s ratio between the adhesive and the adherend. The apparent strength obtained using FW is closer to the local interface strength than the other two types of shear tests but still shows that the apparent strength does not account for the combined stress state.

#### 3.2.2. Effect of Adhesive Thickness on Stress State at Adhesive Interface in Three Different Bond Strength Tests

**Figure 7:**
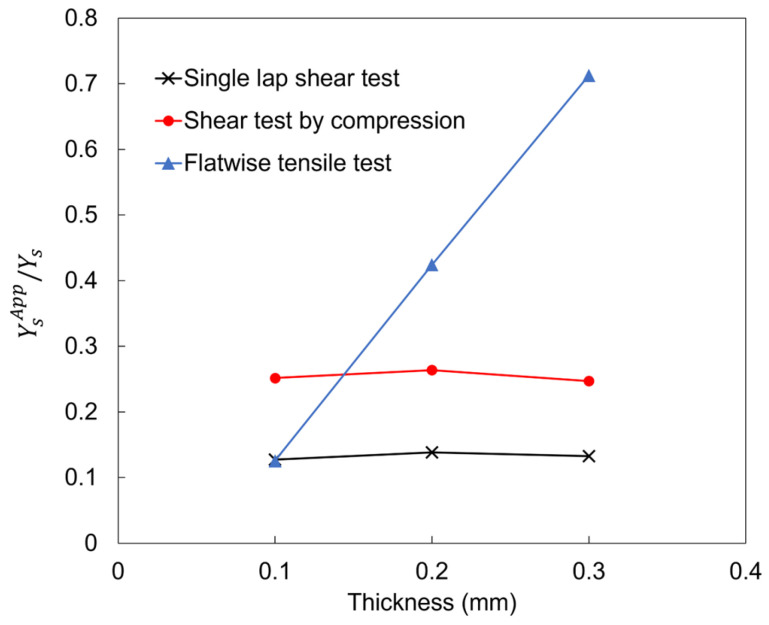
Dependence on weld thickness of the ratio of the apparent pure shear strength to the pure shear strength at the fracture initiation point for three different adhesive thicknesses (0.1, 0.2, and 0.3 mm). The thickness dependence is small in the single-lap shear test and the Shear test by compression, but the thickness of the adhesive (thickness-to-welded area ratio) has a significant effect on the apparent strength in the flatwise tensile test.

The local stress state and apparent strength change at the fracture initiation point for the three types of bond strength tests in relation to the thickness of the adhesive are listed in Table 2. Figure 7 shows a graph comparing the simulation results of the three different tests with $Y_{s}^{App}/Y_{s}$ on the vertical axis and the adhesive thickness on the horizontal axis. The closer the value on the vertical axis is to 1, the smaller the degree of underestimation of apparent strength. Because the reaction force in FW is in the tensile direction toward the adhesive interface, the value on the vertical axis is the ratio of the apparent shear strength to the true shear strength, as predicted by Equation (2). The normal apparent strength of CS and SLS in Table 2 was also determined in the same way outlined in Equation (2).

Focusing on the variation in the $Y_{s}^{App}/Y_{s}$ ratio due to the adhesive thickness, the standard deviations for SLS, SC, and FW were 4.4 × 10^−3^, 7.1 × 10^−3^, and 2.4 × 10^−1^, respectively. SLS and CS are less thickness-dependent but, on average, underestimate the apparent strength to approximately 13.3% and 25.4% of the true strength, respectively. SC is inferior to FW but reduces the discrepancy between the apparent and true strengths more than SLS. FW underestimates the apparent strength by an average of 42.1% of the true bond strength, and the apparent strength reproduces the true bond strength by approximately 71.3% at an adhesive thickness of 0.3 mm. When the adhesive is thin, the rate of shear stress is particularly high and should be used with caution.

## 4. Conclusions

This study proposed a comprehensive method for evaluating the strength of the adhesive–adherend interface of ultrasonically welded specimens by drawing a fracture envelope based on a numerical simulation of the stress state at the fracture initiation point. We have clarified the following points:

1. SLS underestimates the apparent strength to less than 14% of the true strength.
2. FW provides an apparent strength of approximately 42% of the true adhesive strength and has a lower degree of underestimation than the other two shear tests; however, care should be taken because the accuracy of the apparent strength evaluation depends on the adhesive thickness and can vary significantly.
3. A new test method, the shear test using compression (SC), is developed and proves comparable to SLS in terms of adhesive thickness dependence. Although the apparent strength is still underestimated, it is improved to approximately 26% of the true interfacial strength, allowing the test to be performed on smaller specimens.
4. The SC we proposed has the advantages of being able to conduct experiments with small specimens, which makes it easy to apply shear stress to the interface, and of being able to conduct experiments at low cost. However, the amount of data is limited because the fixture is currently in the development stage, and a combination of numerical analyses is still necessary.
5. This paper clarifies the inadequacy of conventional methods for evaluating interface strength in bond strength tests and provides a method for obtaining true interface strength and guidelines for predicting true interface strength from experimental results. This enables accurate numerical simulation of interfacial strength and reliable structural design.

Future considerations include drawing a fracture envelope that accounts for the fracture of the adhesive itself, in addition to the adhesive strength of the adhesive–adherend interface. Under different welding conditions, failure can initiate from material failure rather than from the bonding interface. This approach allows us to determine whether the interface or adhesive will fracture first under specific stress conditions.

## Figures and Tables

**Table 1:** Material properties of CF/PEEK adherend, PEEK adhesive, and interface bonding cohesive.

CF/PEEK [41]	Young modulus (MPa)						Poisson’s ratio		
	E1	E2	E3	G12	G13	G23	Nu12	Nu13	Nu23
	56,800	56,800	8210	43,600	3000	3000	0.25	0.35	0.35
PEEK	Young modulus (MPa)						Poisson’s ratio		
	3000						0.37		
Cohesive	Interfacial strength (MPa)								
	$Y_{n}$						$Y_{s}$		
	48.0126						67.9		

**Table 2:** Local stress state and apparent strength variation at fracture initiation point with adhesive thickness in three bond strength tests: SLS (single-lap shear test), SC (shear test using compression), and FW (flatwise tensile test).

Types of Bond Strength Tests	Thickness of Adhesive [mm]	*t_n_* [MPa]	*t_s_* [MPa]	*Y_n_^App^* [MPa]	*Y_s_^App^* [MPa]
SLS	0.1	36.05	33.90	6.12	8.65
	0.2	37.28	32.11	6.64	9.39
	0.3	38.55	30.14	6.37	9.01
CS	0.1	35.25	35.01	12.08	17.08
	0.2	36.69	32.98	12.66	17.90
	0.3	38.48	30.25	11.85	16.76
FW	0.1	44.23	19.06	6.02	8.52
	0.2	41.18	25.62	20.36	28.80
	0.3	40.20	27.39	34.22	48.39

## Data Availability

Data are contained within the article.
